# Supplementary material for: A systematic review of the effect of The Daily Mile™ on children’s physical activity, physical health, mental health, wellbeing, academic performance and cognitive function
Source: PLoS One. 2023 Jan 12;18(1):e0277375. doi: 10.1371/journal.pone.0277375 (PMC9836306; doi:10.1371/journal.pone.0277375)
Supplement: S1 File — (DOCX) [file pone.0277375.s002.docx]

**S2-Screening tool for independent author screening**

|  | Yes | No | Comments |
| --- | --- | --- | --- |
| **Language**  Is the full paper in English? | Go to next question | Exclude |  |
| **Peer review**  Has the paper been peer reviewed? |  | Exclude |  |
| **Type of study**  Is it a protocol study? | Exclude |  |  |
| **Participants**  Are the participants children aged between 4 and 12 years old? |  | Exclude |  |
| **Intervention type**  Is the intervention The Daily Mile, without additional interventions? |  | Exclude |  |
| **Outcomes**  Does the study report at least one outcome of interest (i.e. physical health, psychological wellbeing, academic performance, cognitive functioning or physical activity levels)? |  | Exclude |  |
| Include paper | | | |
