# Supplementary material for: A systematic review of the effect of The Daily Mile™ on children’s physical activity, physical health, mental health, wellbeing, academic performance and cognitive function
Source: PLoS One. 2023 Jan 12;18(1):e0277375. doi: 10.1371/journal.pone.0277375 (PMC9836306; doi:10.1371/journal.pone.0277375)
Supplement: S2 File — (DOCX) [file pone.0277375.s003.docx]

**S3- Search from PsycINFO**

1.exp Elementary Schools/

2.exp School Based Intervention/

3.("school based intervention" or "primary school" or "school based health promotion").mp. [mp=title, abstract, heading word, table of contents, key concepts, original title, tests & measures, mesh word]

4."daily mile".mp. [mp=title, abstract, heading word, table of contents, key concepts, original title, tests & measures, mesh word]

5.exp Physical Fitness/

6.exp Physical Activity/

7.exp Exercise/

8.exp Running/

9.exp Exercise/

10.exp Walking/

11.exp Cognition/

12.exp Academic Achievement/

13.exp Executive Function/

14.exp Well Being/

15.exp Mental Health/

16.exp Physical Health/

17.exp Health/ or exp Child Health/ or exp Public Health/

18.exp "Quality of Life"/

19.("physical fitness" or "physical activit*" or exercis*).mp. [mp=title, abstract, heading word, table of contents, key concepts, original title, tests & measures, mesh word]

20.(run* or walk* or jog* or cognition or "cognitive function" or "academic performance" or "educational achievement" or "executive function").mp. [mp=title, abstract, heading word, table of contents, key concepts, original title, tests & measures, mesh word]

21.("well being" or wellbeing or well-being or "mental health" or psychological or "physical health*" or health* or "Quality of life" or QOL).mp. [mp=title, abstract, heading word, table of contents, key concepts, original title, tests & measures, mesh word]

22.1 or 2 or 3 or 5 or 6 or 7 or 8 or 9 or 10 or 11 or 12 or 13 or 14 or 15 or 16 or 17 or 18 or 19 or 20 or 2123.4 and 2224.

limit 23 to (english language and yr="2012 -Current")
