# Supplementary material for: A systematic review of the effect of The Daily Mile™ on children’s physical activity, physical health, mental health, wellbeing, academic performance and cognitive function
Source: PLoS One. 2023 Jan 12;18(1):e0277375. doi: 10.1371/journal.pone.0277375 (PMC9836306; doi:10.1371/journal.pone.0277375)
Supplement: S3 File — (DOCX) [file pone.0277375.s004.docx]

| **Final Decision** |
| --- |
| **Study ID** |
| **Title** |
| **Location** |
| **Aim of study** |
| **Study design** |
| **Timing** |
| **Control group** |
| **Number of participants** |
| **Male** |
| **Female** |
| **Mean age (SD)** |
| **Other demographics** |
| **Outcome 1** |
| **Assessment** |
| **Measurement tool** |
| **Association being tested** |
| **Results** |
| **Outcome 2** |
| **Assessment** |
| **Measurement tool** |
| **Association being tested** |
| **Results** |
| **Outcome 3** |
| **Assessment** |
| **Measurement tool** |
| **Association being tested** |
| **Results** |
| **Outcome 4** |
| **Assessment** |
| **Measurement tool** |
| **Association being tested** |
| **Results** |
| **Summary** |
| **Notes** |
